# 8-Hydr­oxy-8-phenyl-2,3,7,8-tetra­hydro-6*H*-1,4-dioxino[2,3-*f*]isoindol-6-one

**DOI:** 10.1107/S1600536808003012

**Published:** 2008-02-06

**Authors:** Viktor A. Tafeenko, Leonid A. Aslanov, Mahmud I. Khasanov, Sergei S. Mochalov

**Affiliations:** a Chemistry Department, Moscow State University, 119991 Moscow, Russian Federation

## Abstract

In the title compound, C_16_H_13_NO_4_, the indole system is essentially planar, whereas the dioxane ring adopts a twist conformation. The mol­ecules are linked into chains by —O— H⋯O=C— hydrogen bonds and these chains are linked into rods by means of N—H⋯O hydrogen bonds. Exept for weak C—H⋯O inter­actions between the rods, no other inter­molecular contacts of inter­est are present.

## Related literature

For details of the appropriate nitrile hydrolysis, see: Moorthy & Singhal (2005 ▶).
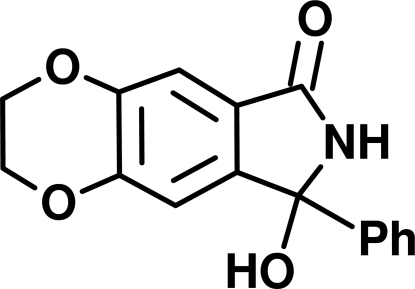


## Experimental

### 

#### Crystal data

C_16_H_13_NO_4_
*M* *_r_* = 283.27
Monoclinic, 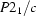

*a* = 8.6001 (17) Å
*b* = 27.005 (5) Å
*c* = 5.7221 (5) Å
β = 92.602 (10)°
*V* = 1327.6 (4) Å^3^
*Z* = 4
Cu *K*α radiation
μ = 0.85 mm^−1^
*T* = 291 (2) K
0.08 × 0.06 × 0.04 mm

#### Data collection

Enraf–Nonius CAD-4 diffractometer
Absorption correction: none
2892 measured reflections
2653 independent reflections
1784 reflections with *I* > 2σ(*I*)
*R*_int_ = 0.025
2 standard reflections frequency: 120 min intensity decay: none

#### Refinement

*R*[*F* ^2^ > 2σ(*F* ^2^)] = 0.054
*wR*(*F* ^2^) = 0.147
*S* = 1.05
2653 reflections
192 parameters
H-atom parameters constrained
Δρ_max_ = 0.23 e Å^−3^
Δρ_min_ = −0.24 e Å^−3^

### 

Data collection: *CAD-4 Software* (Enraf–Nonius, 1989 ▶); cell refinement: *CAD-4 Software*; data reduction: *XCAD4* (Harms & Wocadlo, 1995 ▶); program(s) used to solve structure: *SHELXS97* (Sheldrick, 2008 ▶); program(s) used to refine structure: *SHELXL97* (Sheldrick, 2008 ▶); molecular graphics: *DIAMOND* (Brandenburg, 2000 ▶); software used to prepare material for publication: *WinGX* (Farrugia, 1999 ▶).

## Supplementary Material

Crystal structure: contains datablocks I, global. DOI: 10.1107/S1600536808003012/rk2073sup1.cif

Structure factors: contains datablocks I. DOI: 10.1107/S1600536808003012/rk2073Isup2.hkl

Additional supplementary materials:  crystallographic information; 3D view; checkCIF report

## Figures and Tables

**Table 1:** Hydrogen-bond geometry (Å, °)

*D*—H⋯*A*	*D*—H	H⋯*A*	*D*⋯*A*	*D*—H⋯*A*
O2—H2⋯O3^i^	0.82	1.95	2.725 (3)	158
N7—H7⋯O2^ii^	0.86	2.09	2.922 (3)	161
C5—H5⋯O4^iii^	0.93	2.52	3.404 (3)	160
C19—H19⋯O2	0.93	2.40	2.734 (4)	101

Symmetry codes: (i) 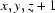; (ii) 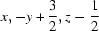; (iii) 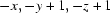.

**Fig. 1.**
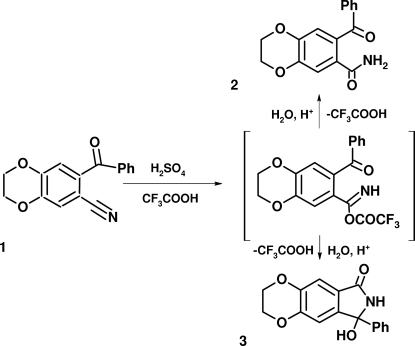
Hydrolysis of (1) did not produce the expected compound, (2) but rather an isomer of (2), viz. compound (3).

**Fig. 2.**
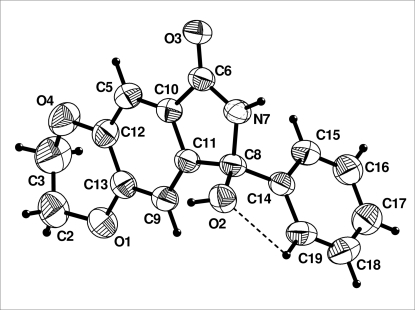
The molecular structure of the title compound, with displacement ellipsoids drawn at the 50% probability level. The dashed line indicates an intramolecular hydrogen bond.

**Fig. 3.**
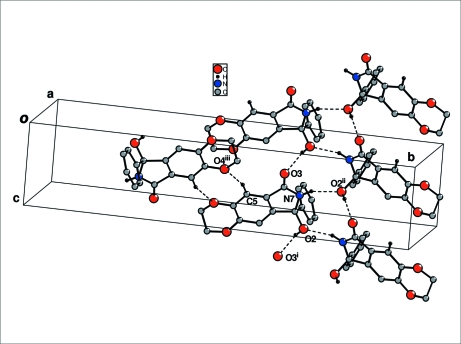
The packing motif of the crystal structure. Hydrogen atoms not involved in hydrogen bonds (dashed lines) have been omitted for clarity. Symmetry codes: (i) x, y, z + 1; (ii) x, -y + 3/2, z - 1/2; (iii) -x, -y + 1, -z + 1.

## supplementary crystallographic information

### Comment

To investigate mechanisms of intra- and intermolecular reactions of *ortho*-substituted benzenes we intended to synthesize novel *ortho*-acyl-substituted benzamides by hydrolysis (Moorthy & Singhal, 2005) of appropriate nitriles. In the case of hydrolysis of 7-benzoyl-2,3-dihydro-1,4-benzodioxine-6-carbonitrile (1) the compound 7-benzoyl-2,3-dihydro-1,4-benzodioxine-6-carboxamide (2) was expected to be produced (Fig. 1). Both the elemental analysis and mass spectroscopic data (*M*+ 283) of the compound we obtained, were in good agreement with structure (2), but ^1^H NMR data were not. Although 13 protons were identified in the ^1^H NMR spectrum, an expected signal for the NH_2_ group was absent. In addition, two single signals were detected in the ^1^H NMR spectrum, each corresponding to one proton of large difference in chemical shift (6.70 and 9.02). To determine the structure of the compound, we carried out an X-ray crystallographic analysis, which revealed that hydrolysis of (1), under the conditions specified by Moorthy & Singhal, did not produce the expected compound (2); instead the product was an isomer of compound (2), *viz*. 8-hydroxy-8-phenyl-2,3,7,8-tetrahydro-6*H*-[1,4]dioxino [2,3-*f*]isoindol-6-one, (3) (Fig. 1).

The dihedral angle between the planes defined by the atoms C5/C9/C10/C11/C12/C13 (plane 1) and C8/N7/C6/C10/C11 (plane 2) (Fig. 2) is 1.64 (9)°. The 6-membered dioxane ring adopts a twist conformation, with atoms C3 and C2 displaced out of plane 1 by 0.375 (4) and -0.273 (3) Å, respectively, compared with displacements of -0.012 (3) and 0.010 (3) Å for O4 and O1, respectively (Fig. 2). The torsion angle O2—C8—C14—C19 has rather a small value [16.7 (3)°]. This results from the intramolecular hydrogen bond C19—H19···O2. The packing motif, as shown in Fig.3, can be described as follows: molecules are linked by hydrogen bonds in head-to-tail fashion through oxy- and keto-groups to form infinite chains. The two adjacent chains are linked by N7—H7···O2^ii^ hydrogen bonds, forming infinite rods running along the *c* axis. Neighbouring rods interact *via* centrosymmetric C5—H5···O4^iii^ hydrogen bonds. Symmetry codes are listed in Table 1.

### Experimental

A mixture of (1) (1 g, 0.0038 mol), concentrated sulfuric acid (1 ml) and trifluoroacetic acid (4 ml) was boiled under reflux. with stirring, for 5 h. The solution was then poured into ice-water (75 ml). The resulting white precipitate was filtered off, washed with water and recrystallized from acetone.

### Refinement

The positions of the H atoms were determined from Fourier difference maps; they were then placed in calculated positions and allowed to ride on their parent atoms [C—H = 0.93–0.97 Å, O—H = 0.82 Å and N—H = 0.86 Å]. *U*_iso_(H) = *xU*_eq_(parent atom), where *x* = 1.5 for attached O and 1.2 for C and N.

### Crystal data

**Table:**

C_16_H_13_NO_4_	*F*_000_ = 592
*M**_r_* = 283.27	*D*_x_ = 1.417 Mg m^−^^3^
Monoclinic, *P*2_1_/*c*	Melting point = 485–486 K
Hall symbol: -P 2ybc	Cu *K*α radiation λ = 1.54184 Å
*a* = 8.6001 (17) Å	Cell parameters from 25 reflections
*b* = 27.005 (5) Å	θ = 26–42º
*c* = 5.7221 (5) Å	µ = 0.85 mm^−^^1^
β = 92.602 (10)º	*T* = 291 (2) K
*V* = 1327.6 (4) Å^3^	Prism, colorless
*Z* = 4	0.08 × 0.06 × 0.04 mm

### Data collection

**Table:**

Enraf–Nonius CAD-4 diffractometer	*R*_int_ = 0.025
Radiation source: fine-focus sealed tube	θ_max_ = 73.9º
Monochromator: graphite	θ_min_ = 3.3º
*T* = 291(2) K	*h* = −10→10
Non–profiled ω scans	*k* = 0→32
Absorption correction: none	*l* = 0→7
2892 measured reflections	2 standard reflections
2653 independent reflections	every 120 min
1784 reflections with *I* > 2σ(*I*)	intensity decay: none

### Refinement

**Table:**

Refinement on *F*^2^	Hydrogen site location: inferred from neighbouring sites
Least-squares matrix: full	H-atom parameters constrained
*R*[*F*^2^ > 2σ(*F*^2^)] = 0.054	*w* = 1/[σ^2^(*F*_o_^2^) + (0.061*P*)^2^ + 0.4104*P*] where *P* = (*F*_o_^2^ + 2*F*_c_^2^)/3
*wR*(*F*^2^) = 0.147	(Δ/σ)_max_ < 0.001
*S* = 1.05	Δρ_max_ = 0.23 e Å^−^^3^
2653 reflections	Δρ_min_ = −0.24 e Å^−^^3^
192 parameters	Extinction correction: SHELXL97 (Sheldrick, 2008), Fc^*^=kFc[1+0.001xFc^2^λ^3^/sin(2θ)]^-1/4^
Primary atom site location: structure-invariant direct methods	Extinction coefficient: 0.0051 (6)
Secondary atom site location: difference Fourier map	

### Special details

**Table:**

Geometry. All e.s.d.'s (except the e.s.d. in the dihedral angle between two l.s. planes) are estimated using the full covariance matrix. The cell e.s.d.'s are taken into account individually in the estimation of e.s.d.'s in distances, angles and torsion angles; correlations between e.s.d.'s in cell parameters are only used when they are defined by crystal symmetry. An approximate (isotropic) treatment of cell e.s.d.'s is used for estimating e.s.d.'s involving l.s. planes.
Refinement. Refinement of *F*^2^ against ALL reflections. The weighted *R*-factor *wR* and goodness of fit *S* are based on *F*^2^, conventional *R*-factors *R* are based on *F*, with *F* set to zero for negative *F*^2^. The threshold expression of *F*^2^ > σ(*F*^2^) is used only for calculating *R*-factors(gt) *etc*. and is not relevant to the choice of reflections for refinement. *R*-factors based on *F*^2^ are statistically about twice as large as those based on *F*, and *R*- factors based on ALL data will be even larger.

### Fractional atomic coordinates and isotropic or equivalent isotropic displacement parameters (Å^2^)

**Table:**

	*x*	*y*	*z*	*U*_iso_*/*U*_eq_	
O1	0.3338 (2)	0.50617 (7)	1.1457 (3)	0.0654 (5)	
O2	0.3093 (2)	0.70474 (6)	0.9980 (3)	0.0549 (5)	
H2	0.2370	0.6906	1.0585	0.082*	
O3	0.1250 (2)	0.65694 (7)	0.3005 (3)	0.0568 (5)	
O4	0.1351 (3)	0.48070 (7)	0.7532 (4)	0.0737 (6)	
N7	0.2877 (2)	0.68919 (8)	0.5922 (3)	0.0498 (5)	
H7	0.2972	0.7182	0.5321	0.060*	
C2	0.2445 (4)	0.46150 (12)	1.1374 (6)	0.0810 (10)	
H2A	0.1463	0.4671	1.2100	0.097*	
H2B	0.3003	0.4360	1.2259	0.097*	
C3	0.2138 (5)	0.44427 (12)	0.8942 (7)	0.0878 (11)	
H3A	0.3117	0.4361	0.8260	0.105*	
H3B	0.1510	0.4144	0.8956	0.105*	
C5	0.1581 (3)	0.56216 (9)	0.6119 (4)	0.0536 (6)	
H5	0.0937	0.5541	0.4826	0.064*	
C6	0.2011 (3)	0.65333 (9)	0.4881 (4)	0.0467 (6)	
C8	0.3642 (3)	0.67510 (9)	0.8154 (4)	0.0455 (6)	
C9	0.3521 (3)	0.58711 (9)	1.0021 (4)	0.0498 (6)	
H9	0.4163	0.5955	1.1313	0.060*	
C10	0.2183 (3)	0.60935 (9)	0.6404 (4)	0.0467 (6)	
C11	0.3138 (3)	0.62151 (9)	0.8321 (4)	0.0446 (5)	
C12	0.1965 (3)	0.52745 (10)	0.7811 (5)	0.0542 (6)	
C13	0.2925 (3)	0.53960 (9)	0.9759 (4)	0.0510 (6)	
C14	0.5399 (3)	0.68000 (9)	0.8127 (4)	0.0458 (6)	
C15	0.6197 (3)	0.66247 (10)	0.6231 (4)	0.0555 (6)	
H15	0.5636	0.6499	0.4937	0.067*	
C16	0.7790 (3)	0.66338 (11)	0.6228 (5)	0.0645 (7)	
H16	0.8301	0.6514	0.4946	0.077*	
C17	0.8631 (4)	0.68214 (12)	0.8136 (5)	0.0683 (8)	
H17	0.9712	0.6829	0.8143	0.082*	
C18	0.7876 (3)	0.69959 (11)	1.0011 (5)	0.0678 (8)	
H18	0.8449	0.7121	1.1297	0.081*	
C19	0.6268 (3)	0.69889 (10)	1.0022 (4)	0.0561 (7)	
H19	0.5767	0.7112	1.1307	0.067*	

### Atomic displacement parameters (Å^2^)

**Table:**

	*U*^11^	*U*^22^	*U*^33^	*U*^12^	*U*^13^	*U*^23^
O1	0.0765 (13)	0.0525 (11)	0.0666 (12)	−0.0020 (9)	−0.0039 (10)	0.0182 (9)
O2	0.0654 (12)	0.0516 (10)	0.0488 (10)	−0.0046 (8)	0.0138 (8)	−0.0083 (8)
O3	0.0575 (11)	0.0718 (12)	0.0406 (9)	−0.0020 (9)	−0.0038 (8)	0.0054 (8)
O4	0.0847 (15)	0.0480 (11)	0.0870 (15)	−0.0136 (10)	−0.0131 (12)	0.0040 (10)
N7	0.0603 (13)	0.0463 (11)	0.0421 (10)	−0.0032 (9)	−0.0049 (9)	0.0071 (9)
C2	0.097 (3)	0.0529 (18)	0.093 (2)	−0.0106 (16)	0.0003 (19)	0.0230 (17)
C3	0.107 (3)	0.0513 (18)	0.104 (3)	−0.0006 (18)	−0.011 (2)	0.0065 (18)
C5	0.0578 (15)	0.0547 (15)	0.0480 (13)	−0.0073 (12)	−0.0020 (11)	−0.0051 (12)
C6	0.0464 (13)	0.0567 (15)	0.0371 (11)	0.0029 (11)	0.0021 (10)	0.0008 (10)
C8	0.0575 (14)	0.0446 (13)	0.0343 (11)	−0.0017 (11)	−0.0001 (10)	−0.0005 (9)
C9	0.0564 (14)	0.0511 (14)	0.0417 (12)	−0.0030 (11)	−0.0005 (10)	0.0034 (11)
C10	0.0512 (13)	0.0500 (14)	0.0388 (11)	−0.0035 (11)	0.0017 (10)	−0.0004 (10)
C11	0.0505 (13)	0.0459 (13)	0.0373 (12)	−0.0014 (10)	0.0022 (10)	0.0010 (10)
C12	0.0608 (16)	0.0454 (14)	0.0565 (15)	−0.0078 (12)	0.0027 (12)	−0.0041 (12)
C13	0.0556 (14)	0.0469 (14)	0.0506 (13)	−0.0001 (11)	0.0042 (11)	0.0074 (11)
C14	0.0567 (14)	0.0436 (13)	0.0370 (11)	−0.0025 (11)	0.0011 (10)	0.0021 (10)
C15	0.0610 (16)	0.0649 (17)	0.0407 (12)	−0.0025 (13)	0.0027 (11)	−0.0039 (12)
C16	0.0606 (16)	0.074 (2)	0.0594 (17)	0.0007 (14)	0.0109 (13)	−0.0030 (15)
C17	0.0570 (16)	0.076 (2)	0.0716 (19)	−0.0002 (14)	0.0011 (14)	0.0022 (16)
C18	0.0635 (18)	0.076 (2)	0.0624 (17)	−0.0039 (15)	−0.0152 (14)	−0.0050 (15)
C19	0.0672 (17)	0.0599 (16)	0.0404 (12)	0.0001 (13)	−0.0040 (11)	−0.0032 (11)

### Geometric parameters (Å, °)

**Table:**

O1—C13	1.362 (3)	C8—C11	1.515 (3)
O1—C2	1.430 (4)	C8—C14	1.518 (3)
O2—C8	1.415 (3)	C9—C11	1.374 (3)
O2—H2	0.8200	C9—C13	1.387 (3)
O3—C6	1.236 (3)	C9—H9	0.9300
O4—C12	1.375 (3)	C10—C11	1.380 (3)
O4—C3	1.424 (4)	C12—C13	1.396 (3)
N7—C6	1.344 (3)	C14—C19	1.386 (3)
N7—C8	1.460 (3)	C14—C15	1.393 (3)
N7—H7	0.8600	C15—C16	1.370 (4)
C2—C3	1.480 (5)	C15—H15	0.9300
C2—H2A	0.9700	C16—C17	1.378 (4)
C2—H2B	0.9700	C16—H16	0.9300
C3—H3A	0.9700	C17—C18	1.363 (4)
C3—H3B	0.9700	C17—H17	0.9300
C5—C12	1.376 (4)	C18—C19	1.384 (4)
C5—C10	1.382 (3)	C18—H18	0.9300
C5—H5	0.9300	C19—H19	0.9300
C6—C10	1.477 (3)		
			
C13—O1—C2	114.4 (2)	C13—C9—H9	120.9
C8—O2—H2	109.5	C11—C10—C5	121.3 (2)
C12—O4—C3	113.5 (2)	C11—C10—C6	108.5 (2)
C6—N7—C8	114.7 (2)	C5—C10—C6	130.2 (2)
C6—N7—H7	122.6	C9—C11—C10	121.1 (2)
C8—N7—H7	122.6	C9—C11—C8	129.1 (2)
O1—C2—C3	111.7 (3)	C10—C11—C8	109.8 (2)
O1—C2—H2A	109.3	O4—C12—C5	117.7 (2)
C3—C2—H2A	109.3	O4—C12—C13	121.2 (2)
O1—C2—H2B	109.3	C5—C12—C13	121.0 (2)
C3—C2—H2B	109.3	O1—C13—C9	116.9 (2)
H2A—C2—H2B	107.9	O1—C13—C12	122.7 (2)
O4—C3—C2	112.1 (3)	C9—C13—C12	120.4 (2)
O4—C3—H3A	109.2	C19—C14—C15	117.8 (2)
C2—C3—H3A	109.2	C19—C14—C8	121.7 (2)
O4—C3—H3B	109.2	C15—C14—C8	120.3 (2)
C2—C3—H3B	109.2	C16—C15—C14	121.5 (3)
H3A—C3—H3B	107.9	C16—C15—H15	119.2
C12—C5—C10	117.9 (2)	C14—C15—H15	119.2
C12—C5—H5	121.0	C15—C16—C17	119.6 (3)
C10—C5—H5	121.0	C15—C16—H16	120.2
O3—C6—N7	126.1 (2)	C17—C16—H16	120.2
O3—C6—C10	127.7 (2)	C18—C17—C16	119.9 (3)
N7—C6—C10	106.23 (19)	C18—C17—H17	120.0
O2—C8—N7	110.3 (2)	C16—C17—H17	120.0
O2—C8—C11	112.82 (19)	C17—C18—C19	120.7 (3)
N7—C8—C11	100.69 (18)	C17—C18—H18	119.7
O2—C8—C14	108.89 (19)	C19—C18—H18	119.7
N7—C8—C14	112.20 (19)	C18—C19—C14	120.4 (3)
C11—C8—C14	111.8 (2)	C18—C19—H19	119.8
C11—C9—C13	118.2 (2)	C14—C19—H19	119.8
C11—C9—H9	120.9		
			
C13—O1—C2—C3	40.4 (4)	C3—O4—C12—C5	162.9 (3)
C12—O4—C3—C2	45.2 (4)	C3—O4—C12—C13	−18.1 (4)
O1—C2—C3—O4	−57.6 (4)	C10—C5—C12—O4	179.4 (2)
C8—N7—C6—O3	179.2 (2)	C10—C5—C12—C13	0.3 (4)
C8—N7—C6—C10	−1.7 (3)	C2—O1—C13—C9	167.3 (3)
C6—N7—C8—O2	−117.5 (2)	C2—O1—C13—C12	−13.5 (4)
C6—N7—C8—C11	1.8 (3)	C11—C9—C13—O1	179.3 (2)
C6—N7—C8—C14	120.9 (2)	C11—C9—C13—C12	0.1 (4)
C12—C5—C10—C11	−0.1 (4)	O4—C12—C13—O1	1.5 (4)
C12—C5—C10—C6	178.4 (3)	C5—C12—C13—O1	−179.5 (2)
O3—C6—C10—C11	179.9 (2)	O4—C12—C13—C9	−179.3 (2)
N7—C6—C10—C11	0.8 (3)	C5—C12—C13—C9	−0.4 (4)
O3—C6—C10—C5	1.3 (4)	O2—C8—C14—C19	16.7 (3)
N7—C6—C10—C5	−177.8 (3)	N7—C8—C14—C19	139.1 (2)
C13—C9—C11—C10	0.2 (4)	C11—C8—C14—C19	−108.6 (3)
C13—C9—C11—C8	−178.9 (2)	O2—C8—C14—C15	−167.4 (2)
C5—C10—C11—C9	−0.2 (4)	N7—C8—C14—C15	−45.1 (3)
C6—C10—C11—C9	−178.9 (2)	C11—C8—C14—C15	67.2 (3)
C5—C10—C11—C8	179.0 (2)	C19—C14—C15—C16	0.5 (4)
C6—C10—C11—C8	0.3 (3)	C8—C14—C15—C16	−175.6 (2)
O2—C8—C11—C9	−64.5 (3)	C14—C15—C16—C17	−0.2 (4)
N7—C8—C11—C9	178.0 (2)	C15—C16—C17—C18	0.1 (5)
C14—C8—C11—C9	58.6 (3)	C16—C17—C18—C19	−0.3 (5)
O2—C8—C11—C10	116.3 (2)	C17—C18—C19—C14	0.5 (5)
N7—C8—C11—C10	−1.2 (3)	C15—C14—C19—C18	−0.6 (4)
C14—C8—C11—C10	−120.5 (2)	C8—C14—C19—C18	175.3 (2)

### Hydrogen-bond geometry (Å, °)

**Table:**

*D*—H···*A*	*D*—H	H···*A*	*D*···*A*	*D*—H···*A*
O2—H2···O3^i^	0.82	1.95	2.725 (3)	158
N7—H7···O2^ii^	0.86	2.09	2.922 (3)	161
C5—H5···O4^iii^	0.93	2.52	3.404 (3)	160
C19—H19···O2	0.93	2.40	2.734 (4)	101

Symmetry codes: (i) *x*, *y*, *z*+1; (ii) *x*, −*y*+3/2, *z*−1/2; (iii) −*x*, −*y*+1, −*z*+1.
